# Risk of cardiac rupture among elderly patients with diabetes presenting with first acute myocardial infarction

**DOI:** 10.3389/fendo.2023.1239644

**Published:** 2023-09-19

**Authors:** Xiaolin Zu, Yanyan Jin, Yaping Zeng, Peng Li, Hai Gao

**Affiliations:** ^1^ Department of Cardiology, Emergency Coronary Unit, Beijing Anzhen Hospital, Capital Medical University, Beijing, China; ^2^ The Key Laboratory of Geriatrics, Beijing Institute of Geriatrics, Institute of Geriatric Medicine, Chinese Academy of Medical Sciences, Beijing Hospital/National Center of Gerontology of National Health Commission, Beijing, China

**Keywords:** acute ST-segment elevated myocardial infarction, cardiac rupture, advanced age, diabetes, risk factor

## Abstract

**Objective:**

We aimed to analyze the risk of cardiac rupture (CR) in aged diabetic patients with acute ST-segment elevated myocardial infarction (STEMI) who were followed up for one month, and analyze its independent risk factors.

**Methods:**

A total of 3063 aged patients with first onset STEMI admitted to Beijing Anzhen Hospital from January 2001 to December 2020 were retrospectively included. There were 2020 patients without diabetes mellitus (DM) and 1043 patients with DM. We used propensity scores matching (PSM) method to balance baseline exposure factors between patients with or without DM, and all were divided the DM group (1043 cases) and the non-DM group (1043 cases) after the PSM. The primary outcome was CR (the composite rate of papillary muscle rupture, ventricular septum perforation, free wall rupture), which was diagnosed based on clinical manifestations and/or echocardiographic findings. Kaplan-meier survival analyses and log-rank test was used to evaluate the risk of CR between the two groups, and *Cox* regression analysis was used to evaluate the independent risk factors for CR.

**Results:**

After PSM, the baseline clinical data were similar between the DM and non-DM group (all *P*>0.05). However, level of glycated hemoglobin was significantly higher in the DM group (*P*<0.05). During 1 month of follow-up, there were 55 (2.64%) cases of CR, most occurred within 48h after admission (40 cases). Among the 55 cases, 11(0.53%) had papillary muscle rupture, 18(0.86%) had ventricular septum perforation, and 26(1.25%) had free wall rupture. Kaplan-meier survival analyses detected that the DM group was associated with significantly increased risk of CR (3.36% *vs.* 1.92%, *HR*=1.532, 95% *CI*: 1.054-2.346, *P*=0.030), ventricular septum perforation (1.05% *vs.* 0.67%, *HR*=1.464, 95% *CI*: 1.021-2.099, *P*=0.038) and free wall rupture (1.63% *vs.* 0.86%, *HR*=1.861, 95% *CI*: 1.074-3.225, *P*=0.027) than those in the non-DM group. Among the 2031 aged STEMI patients without CR, 144 cases (6.90%, 144/2086) died; and among the 55 patients with CR, 37 cases (1.77%, 37/2086) died due to CR. Therefore, twenty percent (20.44%, 37/181) of death was due to CR. Multivariate *Cox* regression analysis indicated that DM (*HR*=1.532, 95%*CI*: 1.054-2.346), age (*HR*=1.390, 95%*CI*: 1.079-1.791), female (*HR*=1.183, 95%*CI*: 1.049-1.334), troponin I (*HR*=1.364, 95%*CI*: 1.108-1.679), brain natriuretic peptide (*HR*=1.512, 95%*CI*: 1.069-2.139), revascularization (*HR*=0.827, 95%*CI*: 0.731-0.936) and β-receptor blocker (*HR*=0.849, 95%*CI*: 0.760-0.948) were independent risk factors of CR (all *P*<0.05).

**Conclusion:**

DM as well as a few other factors, are independent determinants of CR. CR is not a rare event among the aged STEMI patients and twenty percent of deaths are due to CR. However, large sample-sized studies are warranted to confirm these findings.

## Introduction

Cardiac rupture (CR) has become a major clinical problem in patients with acute ST-segment elevated myocardial infarction (STEMI) (1). CR secondary to STEMI is one of the main causes of early mortality, and its in-hospital mortality is more than 50% (2, 3). CR usually manifests as sudden death, and patients without sudden death also involve multiple organ ischemic injury (4). Therefore, CR is a catastrophic complication of STEMI, with an incidence of 1% - 2% (5–7). In the past 40 years, the incidence of CR has generally shown a downward trend. The widespread use of thrombolytic therapy has reduced the mortality by 40% (8). Since the early 1990s, with the development of primary percutaneous coronary intervention (PCI), the short-term and long-term mortality of STEMI has further decreased (9, 10). In the past 20 years, primary PCI has become the preferred reperfusion strategy, and the focus of of STEMI has also shifted from the choice of reperfusion methods to the improvement of medical quality. These progress has also further reduced the incidence of CR (7, 11).

However, the improvement of medical quality in STEMI was not improve the clinical prognosis of patients with CR. Recently, the use of mechanical circulatory support has increased, new interventional therapies have been applied, and surgical techniques have been improved in the treatment of CR, but the mortality remains high (11–13). Recent studies in our center showed that the in-hospital mortality rate of ventricular free wall rupture is still as high as 90% (4).

Whether diabetes affects the risk of CR in elderly STEMI patients remains controversial. The occurrence of CR is affected by many factors, including general conditions, clinical manifestations, laboratory tests and treatment. Old age, female, first myocardial infarction and infarcted myocardial area are risk factors for CR, while reperfusion, early usage of β receptor blockers, angiotensin converting enzyme inhibitor (ACEI)/angiotensin II receptor blocker (ARB) are protective factors of CR. Based on the analysis of 6712 STEMI patients in our research center, we found the neutrophil count ≥ 9 × 10^9^/L was an independent risk factor for ventricular free wall rupture, but DM was not an risk factor for CR. Diabetes is a common complication among elderly STEMI patients, which will significantly increase the risk of adverse cardiovascular events (14). Recently, we retrospectively analyzed a total of 10284 patients with STEMI admitted to Beijing Anzhen Hospital from January 2012 to March 2015. Among them, 81 were diagnosed with CR, including 67 cases of acute left ventricular free wall rupture and 14 cases of ventricular septum perforation. Although patients with CR were associated with higher levels of fasting blood glucose, the incidence rate of DM was similar between the two groups. Logistic regression analysis did not detect any significant relationship between DM and CR, and those with advanced age, recurrent myocardial infarction, low systolic blood pressure, left anterior descending artery disease, decreased hemoglobin, low total protein, and high blood magnesium were prone to have CR(all *P*<0.05) (15). However, patients with diabetes can not only affect wound healing after trauma, but also affect the effect of coronary artery reperfusion, which is strongly related to the occurrence of CR (16). Therefore, whether DM was an independent risk factor of CR remains inconclusive.

We aimed to analyze the risk of CR in aged diabetic patients with STEMI who admitted to Beijing Anzhen Hospital from January 2013 to December 2020 and were followed up for one month, and also evaluate the association between DM and CR.

## Methods

### Patients

This is a retrospective cohort study. A total of 3063 aged patients with STEMI who were consecutively admitted to Beijing Anzhen Hospital from January 2013 to December 2020 were included. There were 2020 patients without DM and 1043 patients with DM. According to the propensity score matching method, all were divided into the DM group (n=1043) and the non-DM group (n=1043).

Inclusion criteria: (1) aged 60-87 years old; (2) diagnosed as STEMI based on clinical manifestations, myocardial enzymes (CK-MB, cTNI/cTNT) and ECG results (17); (3) had complete clinical data and laboratory examination data; (4) had complete echocardiographic findings performed in-hospital and within one-month follow-up after discharge; (5) with the permission of patient.

Exclusion criteria: (1) had old myocardial infarction; (2) had congenital heart disease before admission; (3) combined with aortic dissection; (4) had severe lung, liver and kidney function failure; (5) had serious infection; (6) had malignant tumor, with expected survival time of less than 1 year; (7) had unclear echocardiographic findings; (8) had incomplete clinical data and/or prognosis.

This study meets the requirements of medical ethics and has been approved by the ethics committee of Beijing Anzhen Hospital (ethics number: KS2022068).

### Data collection

We searched the HIS electronic medical record system database of our hospital and extract patients’ data recorded within 24h after admission, including basic demograpic information (age, sex, body mass index [BMI], current smoking), past medical history (duration of diabetes, hypertension, hyperlipidemia, transient ischemic attack or stroke, atrial fibrillation, peripheral artery disease, chronic kidney disease), hemodynamic parameters (heart rate and blood pressure at admission), and blood biochemical test performed within 24h after admission (fasting blood glucose [FBG], glycated hemoglobin [HbA1c], hemoglobin, white blood cell [WBC] count, platelet count, serum creatinine, urea nitrogen, brain natriuretic peptide [BNP], cardiac troponin I [TNI]), transthoracic echocardiographic findings (left ventricular ejection fraction [LVEF], left ventricular end-systolic diameter [LVESd], left ventricular end-diastolic diameter [LVEDd]), coronary angiographic findings (left main artery or three branch lesions, Gensini score), revascularization method (thrombolysis, percutaneous coronary intervention, coronary artery bypass grafting) and drug treatment (dual antiplatelet therapy, warfarin, new oral anticoagulant [NOAC], ACEI, ARB, angiotensin receptor neprilysin inhibitor (ARNI), β-receptor blockers, statins).

### Gensini score

The patients’ coronary artery stenosis was judged according to the Gensini score. All patients underwent quantitative coronary angiography (QCA) or CT angiography coronary angiography (CTA). The results of QCA or CTA were independently analyzed and judged by two professional doctors. The degrees of luminal stenosis <25%, 25%―<50%, 50%―<75%, 75%―<90%, 90%―<99% and 99%―100% were regarded as 1, 2, 4, 8, 16 and 32 points, respectively. Finally, according to the degree of stenosis and the stenosis coefficient, the patient’s Gensini score was calculated (18).

### Diagnosis of CR

Diagnosis of CR was based on clinical manifestations and/or echocardiographic findings. Papillary muscle rupture was suggested by physical examination with a holo-systolic murmur across the precordium, and a transthoracic echocardiogram revealed severe mitral regurgitation, and a transesophageal echocardiogram detected complete disruption of the mitral leaflet, chordal apparatus, and/or the papillary muscle (19). Ventricular free wall rupture was diagnosed by the presence of echo-signal free space of the free wall or presence of pericardial effusion, and Color Doppler showed blood flow shunt between the ventricle and the pericardium when patients developed sudden onset of electro-mechanic dissociation (characterized by cardiogenic shock, conscious disturbance, and pulseless electric activity) (19). Ventricular septum rupture was suggested by physical examination with strong cardiac murmur and diagnosed by echocardiography with presence of echo signal-free pace of the ventricular septum and Color Doppler showed blood flow signal across the ventricular septum (19).

### Main outcome and follow-up

All patients were followed up within one month after admission. The primary outcome was CR (the composite rate of papillary muscle rupture, ventricular septum perforation and free wall rupture), which was diagnosed based on clinical manifestations and/or echocardiographic findings (19).

### Propensity score matching

The matching ratio is 1:1, and the caliper value is 0.2. The patients’ age, female, smoking, hypertension, chronic renal disease, heart rate, diastolic blood pressure, FBG, hemoglobin, WBC count, TNI, BNP, LVEF, Gensini score, revascularization and ACEI/ARB/ARNI were the covariates, and whether CR occurred was the dependent variable.

### Statistical analyses

Descriptive characteristics were summarized as the mean ± standard deviation, medians with interquartile ranges (IQRs), and frequencies with percentages (%), when applicable. Comparisons of continuous variables were analyzed by using t tests, and comparisons of categorical variables were analyzed by using the chi-squared test or Fisher’s exact test. The *Cox* proportional hazards regression model was utilized to analyze the relationship between DM and risk of CR. Multiple potential confounders in this study were also considered, including age, female sex, TNI, BNP, and revascularization. Using the log-rank test, the cumulative incidence of CR was computed by using the Kaplan-Meier survival curve.

All of the statistical analyses were performed by using IBM SPSS software (version 23.0, SPSS Inc., Chicago, IL) and associated packages. Two-tailed *P* values <0.05 were considered to be statistically significant.

## Results

### Baseline clinical data before PSM

As shown in **Table 1**, before PSM, there were 3063 aged STEMI patients, 1573 were women (51.4%), with an average age of 75.1 ± 9.6 years. There were significant differences between the two groups in age, female ratio, BMI, rates of current smoker, peripheral artery disease, heart rate, systolic blood pressure, diastolic blod pressure, fasting blood glucose, HbA1c, hemoglobin, serum creatinine, TNI, BNP, Gensini score, LVEF, LVESd, LVEDd, revascularization rate and the application rate of NOAC, β-receptor blocker, ACEI/ARB/ARNI and statin (all *P*<0.05). Other data were similar between the two groups, and the differences were not statistically significant (*P*>0.05).

**Table 1:** Baseline clinical data between the two groups before PSM.

Characteristic	Non-DM group (n=2020)	DM group (n=1043)	*t/x^2^* value	*P* value
Age(year)	75.3 ± 6.5	74.7 ± 8.8	2.137	0.033
Female(%)	1068(38.9%)	505(48.4%)	5.460	0.019
Body mass index(kg/m^2^)	25.1 ± 3.7	25.5 ± 4.8	2.554	0.011
Current smoker(%)	852(34.2%)	398(38.2%)	4.599	0.032
Duration of diabetes(year)	–	12.1 ± 5.6	–	
Hypertension(%)	531(23.9%)	277(26.6%)	0.026	0.872
Hyperlipidemia(%)	728(37.7%)	349(33.5%)	2.006	0.157
TIA/stroke(%)	156(11.8%)	90(8.6%)	0.765	0.382
Atrial fibrillation(%)	301(14.5%)	139(13.3%)	1.385	0.239
Peripheral artery disease(%)	236(12.4%)	150(14.4%)	4.547	0.033
Chronic kidney disease(%)	286(13.9%)	158(15.1%)	0.544	0.461
Heart rate(bpm)	85.3 ± 10.1	82.7 ± 12.4	6.235	<0.001
Systolic blood pressure(mmHg)	110.7 ± 9.3	113.3 ± 11.4	6.776	<0.001
Didastolic blood pressure(mmHg)	71.4 ± 8.6	72.4 ± 9.5	2.941	0.003
Fasting blood glucose(mmol/L)	6.94 ± 1.62	7.98 ± 3.05	12.325	<0.001
HbA1c(%)	5.42 ± 0.76	6.91 ± 1.40	38.169	<0.001
Hemoglobin(g/dL)	11.0 ± 1.7	11.2 ± 2.5	2.612	0.009
White blood cell(×10^9^/L)	8.94 ± 2.81	10.5 ± 2.9	14.401	<0.001
Platelet count(×10^9^/L)	225.4 ± 28.7	222.8 ± 30.5	2.325	0.020
Serum creatinine(mg/dL)	1.31 ± 0.23	1.37 ± 0.38	5.428	<0.001
Troponin I(ng/ml)	12.7 ± 4.3	14.1 ± 6.0	8.645	<0.001
Brain natriuretic peptide(mmol/L)	360.4 ± 55.4	371.2 ± 95.1	3.965	<0.001
Left main/Three artery(%)	279(12.4%)	149(14.3%)	0.129	0.720
Gensini score	75.2 ± 20.1	76.0 ± 22.5	1.002	0.317
Left ventricular ejection fraction(%)	50.5 ± 4.8	48.5 ± 6.8	9.431	<0.001
LV end-systolic diameter(mm)	38.0 ± 6.0	38.6 ± 7.1	2.460	0.014
LV end-diastolic diameter(mm)	48.7 ± 5.3	49.4 ± 6.4	3.222	0.001
Revascularization(%)	1415(73.4%)	785(75.3%)	9.241	0.002
Dual antiplatelet therapy(%)	1064(52.1%)	512(49.1%)	3.537	0.060
Wafarin(%)	271(13.4%)	150(14.4%)	0.541	0.462
NOAC(%)	116(5.0%)	87(8.3%)	7.507	0.006
β-receptor blocker(%)	340(18.4%)	126(12.1%)	12.037	0.001
ACEI/ARB/ARNI(%)	281(23.6%)	176(16.9%)	4.759	0.029
Statin(%)	817(44.7%)	507(48.6%)	18.682	<0.001

TIA, transient ischemic stroke; HbA1c, glycated hemoglobin; NOAC, new oral anticoagulant; ACEI, angiotensin-converting enzyme inhibitor; ARB, angiotensin II receptor blocker; ARNI, angiotensin receptor neprilysin inhibitor.

### Baseline clinical data after PSM

As shown in **Table 2**, after PSM, there are 2086 aged STEMI patients, including 1006 females (48.2%), with an average age of 74.8 ± 8.7 years. The baseline clinical data were similar between the DM and non-DM group (all *P*>0.05). However, level of HbA1c was significantly higher in the DM group compared with that in the non-DM group (*P*<0.05).

**Table 2:** Baseline clinical data between the two groups after PSM.

Characteristic	Non-DM group (n=1043)	DM group (n=1043)	*t/x^2^* value	*P* value
Age(year)	74.9 ± 8.4	74.7 ± 8.8	0.531	0.596
Female(%)	501(48.0%)	505(48.4%)	0.031	0.861
Body mass index(kg/m^2^)	25.4 ± 4.7	25.5 ± 4.8	0.481	0.631
Current smoker(%)	392(37.6%)	398(38.2%)	0.073	0.787
Duration of diabetes(year)	–	12.1 ± 5.6	–	
Hypertension(%)	263(25.2%)	277(26.6%)	0.489	0.484
Hyperlipidemia(%)	346(33.2%)	349(33.5%)	0.019	0.889
TIA/stroke(%)	83(8.0%)	90(8.6%)	0.310	0.578
Atrial fibrillation(%)	131(12.6%)	139(13.3%)	0.272	0.602
Peripheral artery disease(%)	122(11.7%)	150(14.4%)	0.289	0.591
Chronic kidney disease(%)	147(14.1%)	158(15.1%)	0.465	0.495
Heart rate(bpm)	82.4 ± 11.3	82.7 ± 12.4	0.578	0.564
Systolic blood pressure(mmHg)	112.5 ± 12.7	113.3 ± 11.4	1.514	0.130
Didastolic blood pressure(mmHg)	72.0 ± 10.3	72.4 ± 9.5	0.922	0.357
Fasting blood glucose(mmol/L)	7.91 ± 2.89	7.98 ± 3.05	0.538	0.591
HbA1c(%)	5.23 ± 0.68	6.91 ± 1.40	34.861	<0.001
Hemoglobin(g/dL)	11.0 ± 2.8	11.2 ± 2.5	1.721	0.085
White blood cell(×10^9^/L)	10.7 ± 2.4	10.5 ± 2.9	1.716	0.086
Platelet count(×10^9^/L)	221.5 ± 34.7	222.8 ± 30.5	0.909	0.364
Serum creatinine(mg/dL)	1.34 ± 0.45	1.37 ± 0.38	1.645	0.101
Troponin I(ng/ml)	13.7 ± 5.5	14.1 ± 6.0	1.738	0.082
Brain natriuretic peptide(mmol/L)	364.2 ± 75.3	371.2 ± 95.1	1.864	0.063
Left main/Three artery(%)	140(13.4%)	149(14.3%)	0.325	0.568
Gensini score	75.2 ± 20.7	76.0 ± 22.5	0.845	0.398
Left ventricular ejection fraction(%)	49.0 ± 5.5	48.5 ± 6.8	1.846	0.065
LV end-systolic diameter(mm)	38.1 ± 6.3	38.6 ± 7.1	1.701	0.089
LV end-diastolic diameter(mm)	49.0 ± 5.9	49.4 ± 6.4	1.477	0.139
Revascularization(%)	779(74.7%)	785(75.3%)	0.092	0.762
Dual antiplatelet therapy(%)	504(48.3%)	512(49.1%)	0.123	0.726
Wafarin(%)	141(13.5%)	150(14.4%)	0.324	0.570
NOAC(%)	95(9.1%)	87(8.3%)	0.385	0.535
β-receptor blocker(%)	151(14.5%)	126(12.1%)	1.647	0.199
ACEI/ARB/ARNI(%)	163(15.6%)	176(16.9%)	0.595	0.440
Statin(%)	494(47.4%)	507(48.6%)	0.325	0.569

TIA, transient ischemic stroke; HbA1c, glycated hemoglobin; NOAC, new oral anticoagulant; ACEI, angiotensin-converting enzyme inhibitor; ARB, angiotensin II receptor blocker; ARNI, angiotensin receptor neprilysin inhibitor.

### Rate of CR during follow-up

There were 55 (2.64%) aged STEMI patients with CR, most (40 cases, 72.7%) occurred within 48h after admission, and the median occurrence time was 24h after admission (5h, 96h).

Among the 55 cases of CR, there were 11 cases (0.53%) of papillary muscle rupture, 18 cases (0.86%) of ventricular septal perforation, and 26 cases (1.25%) of ventricular free wall rupture (**Table 3**).

**Table 3:** Rate of CR between the two groups after PSM.

prognosis	Non-DM group (n=1043)	DM group (n=1043)	*Log-rank* *x^2^* value	*P* value
Papillary muscle rupture(%)	4(0.38%)	7(0.67%)	1.638	0.066
Ventricular septum perforation(%)	7(0.67%)	11(1.05%)	2.073	0.038
Free wall rupture(%)	9(0.86%)	17(1.63%)	2.285	0.027
All events (%)	20(1.92%)	35(3.36%)	2.617	0.030

**Figure 1:**
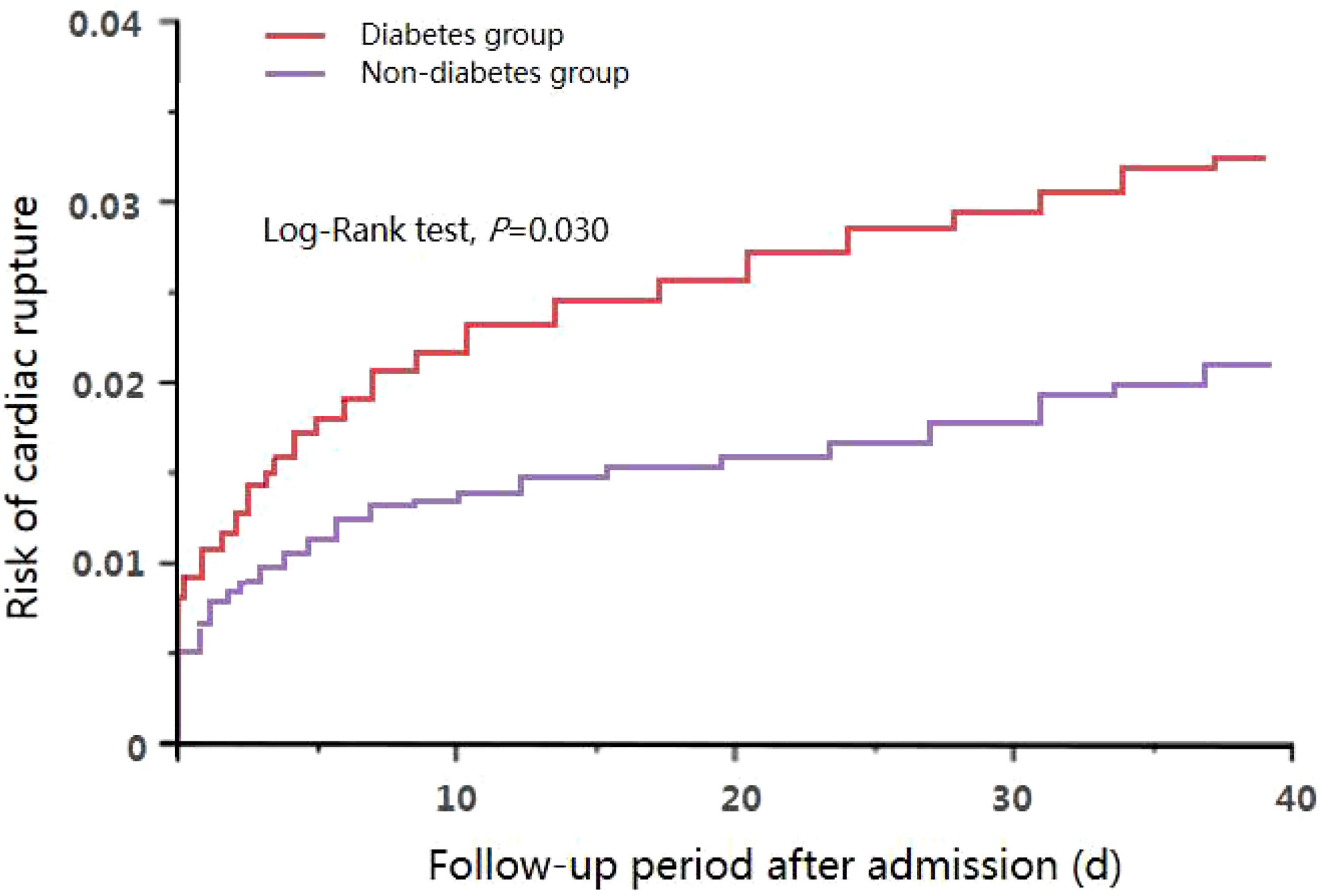
Rate of CRbetween the two groups.

Kaplan-meier survival analysis and log-rank test results showed that the incidence of overall CR (3.36% *vs.* 1.92%, *HR*=1.532, 95% *CI*: 1.054-2.346, *P*=0.030) (**Figure 1**) in the DM group was significantly higher than that in the non-DM group. Additionally, the incidences of ventricular septum perforation (1.05% *vs.* 0.67%, *HR*=1.464, 95% *CI*: 1.021-2.099, *P*=0.038) and free wall rupture (1.63% *vs.* 0.86%, *HR*=1.861, 95% *CI*: 1.074-3.225, *P*=0.027) were significantly increased in the DM group than those in the non-DM. However, the incidence of papillary muscle rupture were similar between the two groups (0.67% *vs.* 0.38%, *HR*=1.315, 95% *CI*: 0.982-1.761, *P*=0.066).

### Survival of patients with STEMI

Among the 2031 aged STEMI patients without CR, 144 cases (6.90%, 144/2086) died; and among the 55 patients with CR, 37 cases (1.77%, 37/2086) died due to CR. Therefore, twenty percent (20.44%, 37/181) of death was due to CR. Of the 11 patients with papillary muscle rupture, 4 cases were completely ruptured, complicated with acute left heart failure, and died after rescue. The remaining 7 cases underwent valve and chordae tendineae repair, and survived after 1 month follow-up. Meanwhile, of the 18 patients with ventricular septum perforation, 5 died due to cardiogenic shock; the remaining 13 patients underwent surgical repair. However, 10 survived and 3 died after operation. Of the 26 patients with free wall rupture, only 1 patient underwent surgery and survived, and the remaining 25 patients died. Therefore, among the 55 patients with CR, 37 cases (67.3%) died after a follow-up of 1 month, most of them had free wall rupture (25 cases) or ventricular septum perforation (8 cases). Therefore, among the 55 cases of CR, only 8 cases (32.7%) survived, and all received cardiac surgery.

### Risk factors of CR

The results of univariate cox regression analysis (**Table 4**) showed that diabetes, age, female, chronic renal disease, heart rate, fasting blood glucose, HbA1c, TNI, WBC count, BNP, serum creatinine, revascularization, β-receptor blocker, ACEI/ARB/ARNI were important risk factors of CR (*P*<0.05).

**Table 4:** Univariate and multivariate Cox regression analyses.

Factors	Univariate			Multivariate			
	HR	95%CI	P value	HR		95%CI	P value
Age	1.432	1.105-1.856	0.007	1.390		1.079-1.791	0.011
Female	1.219	1.063-1.398	0.005	1.183		1.049-1.334	0.006
Chronic kidney disease	1.464	1.093-1.961	0.011	–			
Heart rate	1.218	1.052-1.410	0.008	–			
Fasting blood glucose	1.317	1.061-1.635	0.013	–			
HbA1c	1.255	1.037-1.519	0.020	–			
Cardiac troponin I	1.416	1.142-1.756	0.002	1.364		1.108-1.679	0.003
White blood cell	1.303	1.033-1.644	0.026	–			
Brain natriuretic peptide	1.615	1.108-2.354	0.013	1.512		1.069-2.139	0.019
Serum creatinine	1.481	1.016-2.159	0.041	–			
Revascularization	0.794	0.664-0.950	0.016	0.827		0.731-0.936	0.003
β-receptor blocker(%)	0.834	0.712-0.977	0.024	0.849		0.760-0.948	0.004
ACEI/ARB/ARNI(%)	0.947	0.905-0.991	0.019	–			
Diabetes	1.482	1.079-2.036	0.015	1.532		1.054-2.346	0.030

HbA1c, glycated hemoglobin; ACEI, angiotensin-converting enzyme inhibitor; ARB, angiotensin II receptor blocker; ARNI, angiotensin receptor neprilysin inhibitor.

Multivariate Cox regression analyses (**Table 4**) detected that DM (HR=1.532, 95%CI: 1.054-2.346), age (HR=1.390, 95%CI: 1.079-1.791), female (HR=1.183, 95%CI: 1.049-1.334), TNI (HR=1.364, 95%CI: 1.108-1.679), BNP (HR=1.512, 95%CI: 1.069-2.139), revascularization (HR=0.827, 95%CI: 0.731-0.936), β-receptor blocker (HR=0.849, 95%CI: 0.760-0.948) were independent risk factors of CR (all P<0.05).

## Discussion

At present, although the incidence of CR is significantly reduced, it is still an important cause of death in elderly patients with STEMI. In our study, among the aged patients with STEMI, 55 (2.64%) had CR, which was slightly higher than previous studies. It may be related to the aged patients included in this study. In addition, previous epidemiological studies have detected that CR usually occurred within one week, mostly within 3-5 days after STEMI. Similarly, our study also confirmed that in aged patients with STEMI, CR mostly occurred within 48h after admission. Therefore, in clinical practice, we should also pay attention to the high risk of CR in aged patients with STEMI.

Whether DM was an independent risk factor of CR has been controversial (11, 12). In the APEX-STEMI study, 5745 patients with STEMI were included, with a median age of 68 years. The researchers found 52 cases (0.91%) of CR during follow-up, including 30 cases (0.52%) of free wall rupture, 15 cases (0.26%) of papillary muscle rupture, and 10 cases (0.17%) of ventricular septum perforation. However, they found that there was no significant difference in the incidence of DM betwee patients with or without CR (*P*>0.05). Additionally, multivariate *logistic* regression analyses did not detect any significant association between DM and the risk of CR (*P*>0.05) (13). However, in another study which enrolled 148881 aged patients (67-71 years) with first-onset STEMI, all were divided into the CR group (n=408, with free wall rupture) and the control group (n=148473, without CR). They found that the occurrence of DM history was significantly lower than that in the control group (18% *vs.* 24%), and DM was a protective factor of ventricular free wall rupture (*OR*=0.67,95%*CI*: 0.52-0.87) (14). Meanwhile, for patients with papillary muscle rupture or ventricular septum perforation, diabetes is more rare (15, 16), and several published studies did not detect the association between DM and the risk of papillary muscle rupture or ventricular septum perforation (*P*>0.05). Therefore, Whether DM was a risk factor of CR in aged patients with STEMI is still inconclusive.

We confirmed that DM was associated with increased risk of CR. In our study, 2086 aged patients with first-onset STEMI were included, and 55 patients (2.64%) had CR during 1 month follow-up. We found that DM was associated with significantly increased risk of CR (3.36% *vs.* 1.92%, *HR*=1.532, 95% *CI*: 1.054-2.346, *P*=0.030). Furthermore, the risk of ventricular septum perforation (1.05% *vs.* 0.67%, *HR*=1.464, 95% *CI*: 1.021-2.099, *P*=0.038) and free wall rupture (1.63% *vs.* 0.86%, *HR*=1.861, 95% *CI*: 1.074-3.225, *P*=0.027) were also marked higher in the DM group than those in the non-DM group. However, the incidence of papillary muscle rupture were similar between the two groups (0.67% *vs.* 0.38%, *HR*=1.315, 95% *CI*: 0.982-1.761, *P*=0.066). Therefore, DM as well as a few other factors, were independent determinants of CR.

There were several strengths in our study. First, our study’s sample size was relatively large. We enrolled 3063 aged patients with STEMI who were consecutively admitted to our hospital from January 2013 to December 2020. There were 2020 patients without DM (non-DM group) and 1043 patients with DM (DM group). Second, the baseline clinical data was comparable between those with or wihout DM. Previously, there was no study to specifically compare the risk of CR between the DM and non-DM groups, and there were significant differences in baseline clinical data between those with or without DM. In our study, using the PSM method, patients were divided into the DM group (n=1043) and the non-DM group (n=1043), and the baseline clinical data were almost all comparable between the DM and non-DM group, except the level of HbA1c. Third, only aged patients with first-onset STEMI were included, and those were the high-risk patients for developing CR. Fourth, all patients were followed up for 1 month after admission. Most previous studies only reported the risk of CR occurred in-hospital and many CR cases would be missed. However, large sample-sized studies are warranted to confirm these findings.

Diabetes involved multiple cardiac repair mechanisms after STEMI. Myocardial repair consisted of several stages: (1) early repair stage (within 72h in the early stage); (2) late repair and proliferation stage (within 72h-10d in the early stage); (3) maturation of proliferative tissue and ventricular remodeling stage (lasting for several months) (19, 20). CR mainly occurred in the early and late repair stage, that is, within 7d after the occurrence of STEMI (21). In the first 72h after MI, the myocardial cells in the infarct focus die, leading to the myocardial thinning in this area. At the same time, the release of dead cells in the infarct focus and the lysis of extracellular matrix could generate a large number of risk related molecular signals, which can be captured by the pattern recognition receptor on the immune cells, thus causing the activation of immune cells and infiltration into the infarct focus (22). The infiltrating immune cells not only engulf the cell debris in the infarcted area, but also release various enzymes, further degrading the extracellular matrix and making the myocardium more fragile, and eventually leading to myocardial damage (23). This aseptic inflammatory process can be well controlled by subsequent anti-inflammatory and proliferative processes, and recent studies have found that this processes were closely related to a class of specialized proresolving mediators (SPMs) (24–26). Previous studies have reported that SPM can inhibit the infiltration of pro-inflammatory polylobulated neutrophils into tissues, promote macrophage differentiation towards an anti-inflammatory phenotype, and enhance their ability to phagocytose apoptotic neutrophils. In addition, it had the function of regulating the transition of T cells from an activated state to a regulated state (27). However, in patients with DM, serum SPM and SPM related synthase 15-LOX-1 were significantly lower. In addition, among the DM patients, the inflammatory digestion process in the infarct focus was damaged, which led to the expansion of the ischemic focus (28). Moreover, the content of pro-inflammatory M1 phenotype macrophages was significantly increased in DM patients, and further increased the risk of CR development (29–31).

Many other factors were also associated with the occurrence of CR. Previous studies confirmed that age, medical history, clinical manifestations, biochemical test findings, and treatment (32–34). Qian et al. (34) designed a clinical risk scoring system for predicting CR in STEMI patients. Seven factors were associated with the risk of CR, including age, gender, heart rate, myocardial infarction site, hemoglobin count, white blood cell count, and admission time. Meanwhile, early use of ACEI/ARB and β-receptor blockers could help prevent cardiac rupture. Revascularization, especially the primary PCI should be carried out as soon as possible to minimize the occurrence of CR (35, 36). Similar to previous findings, we found that age (HR=1.390, 95%CI: 1.079-1.791), female (HR=1.183, 95%CI: 1.049-1.334), troponin I (HR=1.364, 95%CI: 1.108-1.679), BNP (HR=1.512, 95%CI: 1.069-2.139), revascularization (HR=0.827, 95%CI: 0.731-0.936) and β-receptor blocker (HR=0.849, 95%CI: 0.760-0.948) were also strongly related to the occurrence of CR (all *P*<0.05). Therefore, considering the extremely high risk of mortality with CR, it is necessary to further confirm its predictive factors, in order to prevent and interven early and timely (37, 38).

## Limitations

There are several limitations in our study. (1) It was conducted in a single center, and the patients’s characteristics maybe different from other centers. (2) Our study’s sample size was not large enough. The risk of CR is relatively low. Therefore, larger sample-sized studies are warranted to confirm the association between DM and CR. (3) CR was diagnosed by echocardiography. However, echocardiography examination is operator dependent, and several patients’ follow-up visits were conducted in non-teaching hospital. Therefore, some CR cases might be missed. (4) The follow-up period was relatively short. Meanwhile, follow-up mainly relied on outpatient services, and some therapeutic information could have been missed, which might affect the incidence of CR (39). Therefore, high qualified studies are needed to confirm these findings.

## Conclusions

DM as well as a few other factors, are independent determinants of CR. CR is not a rare event among the aged STEMI patients. Meanwhile, twenty percent (20.44%, 37/181) of deaths are due to CR. However, large sample-sized studies are warranted to confirm these findings.

## Data availability statement

The raw data supporting the conclusions of this article will be made available by the authors, without undue reservation.

## Ethics statement

The studies involving humans were approved by Beijing Anzhen Hospital Ethics Committee. The studies were conducted in accordance with the local legislation and institutional requirements. The ethics committee/institutional review board waived the requirement of written informed consent for participation from the participants or the participants’ legal guardians/next of kin because This was a retrospective study.

## Author contributions

XZ conducted this study and wrote the paper. YJ, YZ and PL analyzed the data. HG designed this study. All authors contributed to the article and approved the submitted version.

## References

[1] National Center for Cardiovascular Disease. China cardiovascular health and disease report 2021[M]. Beijing: Science Press (2022).

[2] Figueras J Alcalde O Barrabés JA Serra V Alguersuari J Cortadellas J. Changes in hospital mortality rates in 425 patients with acute ST-elevation myocardial infarction and CRover a 30-year period. Circulation (2008) 118(25):2783–9. doi: 10.1161/CIRCULATIONAHA.108.776690 19064683

[3] López-Sendón J Gurfinkel EP Lopez de Sa E Agnelli G Gore JM Steg PG. Factors related to heart rupture in acute coronary syndromes in the Global Registry of Acute Coronary Events. Eur Heart J (2010) 31(12):1449–56. doi: 10.1093/eurheartj/ehq061 20231153

[4] Gong W Shi H Yan M Yan Y Wang X Li S. Clinical manifestation, timing course, precipitating factors, and protective factors of ventricular free wall rupture following ST-segment elevation myocardial infarction. Int Heart J (2020) 61(4):651–7. doi: 10.1536/ihj.19-541 32684590

[5] Gao XM White DA Dart AM Du XJ. Post-infarct cardiac rupture: recent insights on pathogenesis and therapeutic interventions. Pharmacol Ther (2012) 134(2):156–79. doi: 10.1016/j.pharmthera.2011.12.010 22260952

[6] Damluji AA van Diepen S Katz JN Menon V Tamis-Holland JE Bakitas M. Mechanical complications of acute myocardial infarction: A scientific statement from the American Heart Association. Circulation (2021) 144(2):e16–35. doi: 10.1161/CIR.0000000000000985 PMC9364424 34126755

[7] Honda S Asaumi Y Yamane T Nagai T Miyagi T Noguchi T. Trends in the clinical and pathological characteristics of CRin patients with acute myocardial infarction over 35 years. J Am Heart Assoc (2014) 3(5):e000984. doi: 10.1161/JAHA.114.000984 25332178 PMC4323797

[8] ISIS-2 (Second International Study of Infarct Survival) Collaborative Group. Randomised trial of intravenous streptokinase, oral aspirin, both, or neither among 17, 187 cases of suspected acute myocardial infarction: ISIS-2 [J]. Lancet (1988) 2(8607):349–60. doi: 10.1016/S0140-6736(88)92833-4 2899772

[9] Keeley EC Boura JA Grines CL. Primary angioplasty versus intravenous thrombolytic therapy for acute myocardial infarction: a quantitative review of 23 randomised trials. Lancet (2003) 361(9351):13–20. doi: 10.1016/S0140-6736(03)12113-7 12517460

[10] Grines CL Browne KF Marco J Rothbaum D Stone GW O'Keefe J. A comparison of immediate angioplasty with thrombolytic therapy for acute myocardial infarction. The Primary Angioplasty in Myocardial Infarction Study Group. N Engl J Med (1993) 328(10):673–9. doi: 10.1056/NEJM199303113281001 8433725

[11] Puerto E Viana-Tejedor A Martínez-Sellés M Domínguez-Pérez L Moreno G Martín-Asenjo R. Temporal trends in mechanical complications of acute myocardial infarction in the elderly. J Am Coll Cardiol (2018) 72(9):959–66. doi: 10.1016/j.jacc.2018.06.031 30139440

[12] Goldsweig AM Wang Y Forrest JK Cleman MW Minges KE Mangi AA. Ventricular septal rupture complicating acute myocardial infarction: incidence, treatment, and outcomes among medicare beneficiaries 1999-2014. Catheter Cardiovasc Interv (2018) 92(6):1104–15. doi: 10.1002/ccd.27576 29513365

[13] Jones BM Kapadia SR Smedira NG Robich M Tuzcu EM Menon V. Ventricular septal rupture complicating acute myocardial infarction: a contemporary review. Eur Heart J (2014) 35(31):2060–8. doi: 10.1093/eurheartj/ehu248 24970335

[14] Fu W Dong R Zheng JB Zhang K Mu JS. Risk factors of early death and long-term outcomes in myocardial infarction complicated with ventricular septal rupture. Chin J Geriatr (2022) 41(5):517–22. doi: 10.3760/cma.j.issn.0254-9026.2022.05.003

[15] Liu ZJ Miao HT Nie SP. Risk factors of CRin patients with acute myocardial infarction. Chin J Cardiol (2016) 44(10):862–7. doi: 10.3760/cma.j.issn.0253-3758.2016.10.007 27903372

[16] Miao HT Zhang M Liu ZJ Cheng J Chen ZS Nie SP. Clinical characteristics and prognosis of patients with acute myocardial infarction complicated with different parts of heart rupture. Chin Crit Care Med (2016) 28(12):1080–5. doi: 10.3760/cma.j.issn.2095-4352.2016.12.003

[17] Emergency Medical Branch of Chinese Medical Doctor Association Cardiovascular Epidemiology Branch of Chinese Medical Association Laboratory Medicine Branch of Chinese Medical Association. Emergency rapid diagnosis and treatment of guidelines acute coronary syndrome. Chin J Emerg Med (2016) 25(4):397–404. doi: 10.3760/cma.j.issn.1671-0282.2016.04.002

[18] Rampidis GP Benetos G Benz DC Giannopoulos AA Buechel RR. A guide for Gensini Score calculation. Atherosclerosis (2019) 287:181–3. doi: 10.1016/j.atherosclerosis.2019.05.012 31104809

[19] Lu Q Liu P Huo JH Wang YN Ma AQ Yuan ZY. Cardiac rupture complicating acute myocardial infarction: the clinical features from an observational study and animal experiment. BMC Cardiovasc Disord (2020) 20(1):409. doi: 10.1186/s12872-020-01683-y 32912149 PMC7488297

[20] Prabhu SD Frangogiannis NG. The biological basis for cardiac repair after myocardial infarction: from inflammation to fibrosis. Circ Res (2016) 119(1):91–112. doi: 10.1161/circresaha.116.303577 27340270 PMC4922528

[21] Sutton MG Sharpe N. Left ventricular remodeling after myocardial infarction: pathophysiology and therapy. Circulation (2000) 101(25):2981–8. doi: 10.1161/01.cir.101.25.2981 10869273

[22] Gong FF Vaitenas I Malaisrie SC Maganti K. Mechanical complications of acute myocardial infarction: A review. JAMA Cardiol (2021) 6(3):341–9. doi: 10.1001/jamacardio.2020.3690 33295949

[23] Timmers L Pasterkamp G de Hoog VC. The innate immune response in reperfused myocardium. Cardiovasc Res (2012) 94(2):276–83. doi: 10.1093/cvr/cvs018 22266751

[24] Hess A Borchert T Ross TL. Characterizing the transition from immune response to tissue repair after myocardial infarction by multiparametric imaging. Basic Res Cardiol (2022) 117(1):14. doi: 10.1007/s00395-022-00922-x 35275268 PMC8917105

[25] Fosshaug LE Colas RA Anstensrud AK Gregersen I Nymo S Sagen EL. Early increase of specialized pro-resolving lipid mediators in patients with ST-elevation myocardial infarction. EBioMedicine (2019) 46:264–73. doi: 10.1016/j.ebiom.2019.07.024 PMC6711324 31345784

[26] Serhan CN Levy BD. Resolvins in inflammation: emergence of the pro-resolving superfSTEMIly of mediators[J]. J Clin Invest (2018) 128(7):2657–69. doi: 10.1172/jci97943 PMC6025982 29757195

[27] Chiurchiù V Leuti A Dalli J Jacobsson A Battistini L Maccarrone M. Proresolving lipid mediators resolvin D1, resolvin D2, and maresin 1 are critical in modulating T cell responses. Sci Transl Med (2016) 8(353):353ra111. doi: 10.1126/scitranslmed.aaf7483 PMC5149396 27559094

[28] Tang X Liu L Miao Z Zhang J Cai X Zhao BQ. Resolution of inflammation is disturbed in acute ischemic stroke with diabetes mellitus and rescued by resolvin D2 treatment. Free Radic Biol Med (2022) 188:194–205. doi: 10.1016/j.freeradbiomed.2022.06.231 35750271

[29] Heilbronn LK Campbell LV. Adipose tissue macrophages, low grade inflammation and insulin resistance in human obesity. Curr Pharm Des (2008) 14(12):1225–30. doi: 10.2174/138161208784246153 18473870

[30] Okonkwo UA DiPietro LA. Diabetes and wound angiogenesis. Int J Mol Sci (2017) 18(7):1419. doi: 10.3390/ijms18071419 28671607 PMC5535911

[31] Sawaya AP Stone RC Brooks SR Pastar I Jozic I Hasneen K. Deregulated immune cell recruitment orchestrated by FOXM1 impairs human diabetic wound healing. Nat Commun (2020) 11(1):4678. doi: 10.1038/s41467-020-18276-0 32938916 PMC7495445

[32] Al-Masawa ME Alshawsh MA Ng CY Ng AMH Foo JB Vijakumaran U. Efficacy and safety of small extracellular vesicle interventions in wound healing and skin regeneration: A systematic review and meta-analysis of animal studies. Theranostics (2022) 12(15):6455–508. doi: 10.7150/thno.73436 PMC9516230 36185607

[33] Oualha D Ben Abderrahim S Ben Abdeljelil N BelHadj M Ben Jomâa S Saadi S. CRduring acute myocardial infarction : Autopsy study (2004-2020). Ann Cardiol Angeiol (Paris) (2023) 72(3):101601. doi: 10.1016/j.ancard.2023.101601 37060875

[34] Qian G Jin RJ Fu ZH Yang YQ Su HL Dong W. Development and validation of clinical risk score to predict the CRin patients with STEMI. Am J Emerg Med (2017) 35(4):589–93. doi: 10.1016/j.ajem.2016.12.033 28132793

[35] David TE. Post-infarction ventricular septal rupture. Ann Cardiothorac Surg (2022) 11(3):261–7. doi: 10.21037/acs-2021-STEMI-111 PMC9207689 35733715

[36] Simsek B Kostantinis S Karacsonyi J Brilakis ES. Can we predict CRin patients with ST-segment elevation myocardial infarction? J Thorac Dis (2022) 14(7):2451–3. doi: 10.21037/jtd-22-655 PMC9344428 35928604

[37] De Lazzari M Cipriani A Cecere A Niero A De Gaspari M Giorgi B. CRin acute myocardial infarction: a cardiac magnetic resonance study. Eur Heart J Cardiovasc Imaging (2023), jead088. doi: 10.1093/ehjci/jead088 37200615 PMC10610764

[38] Xu Z Li Y Zhang R Liu Y Liu H Yu J. Risk factors for CRafter acute ST-segment elevation myocardial infarction during the percutaneous coronary intervention era: a retrospective case-control study. J Thorac Dis (2022) 14(4):1256–66. doi: 10.21037/jtd-22-394 PMC9096283 35572908

[39] Yousef S Sultan I VonVille HM Kahru K Arnaoutakis GJ. Surgical management for CRof acute myocardial infarction: a systematic review of long-term outcomes. Ann Cardiothorac Surg (2022) 11(3):239–51. doi: 10.21037/acs-2021-STEMI-20 PMC9207694 35733723

